# COVID-Induced Thrombotic Thrombocytopenic Purpura: A Case Report and Treatment-Focused Review

**DOI:** 10.7759/cureus.57252

**Published:** 2024-03-30

**Authors:** Madeeha Subhan Waleed, Lohitha Dhulipalla, Muhammad Niazi, Terenig Terjanian, Meekoo Dhar

**Affiliations:** 1 Internal Medicine, Lower Bucks Hospital, Bristol, USA; 2 Hematology and Oncology, Northwell Health, Staten Island, USA; 3 Internal Medicine, Northwell Health/Staten Island University Hospital, Staten Island, USA; 4 Hematology and Oncology, Staten Island University Hospital, Staten Island, USA

**Keywords:** caplacizumab, rituximab therapy, refractory thrombocytopenia, thrombotic thrombocytopenic thrombocytopenia, thrombotic microangiopathy (tma), acquired ttp, covid-19

## Abstract

Thrombotic thrombocytopenic purpura (TTP) is a rare disease that is part of a vast spectrum of thrombotic microangiopathies (TMAs). Despite the rarity of TTP, clinicians must maintain a high suspicion of this disease. The condition is characterized by fever, low platelets, hemolytic anemia, renal abnormalities, and neurological dysfunction. However, all these symptoms are not necessarily present in all the patients. In this review, we describe a case of a 51-year-old female who presented to the emergency department (ED) with chief complaints of dizziness and lightheadedness, subsequently leading to a diagnosis of TTP, caused as a result of COVID-19. This review raises awareness so that there is early recognition of any hematological manifestations associated with COVID-19, reducing the morbidity and mortality associated with the disease. Due to the unpredictability of COVID-19 and its complications, robust research is needed to understand the mechanism and determine which patients are more at risk for adverse outcomes.

## Introduction

Thrombotic thrombocytopenic purpura (TTP) is a rare disease that is part of a vast spectrum of thrombotic microangiopathies (TMAs). TTP presents more commonly in females, particularly in the fifth decade of life [1]. Despite the rarity of TTP, clinicians must maintain a high suspicion of this disease, as untreated TTP carries a mortality rate of up to 90% [2]. The condition is characterized by fever, low platelets, hemolytic anemia, renal abnormalities, and neurological dysfunction. However, all these symptoms are not necessarily present in all the patients [3]. TTP results from ADAMTS-13 deficiency leading to ultra-large von Willebrand factor-microthrombi formation within the vasculature resulting in ischemia of the tissue and end-organ damage [3]. Organ ischemia is related to the widespread microthrombi and can involve the brain, heart, and less frequently mesenteric vasculature and kidney [4]. The more prevalent findings that raise suspicion for TTP are profound thrombocytopenia (usually less than 30,000 x 10/l) and microangiopathic hemolytic anemia (with schistocytes in a blood smear); both are associated with their relative signs: cutaneous and mucosal bleeding, weakness, and dyspnea [1]. The incidence of TTP is three in one million adults per year [5]. TTP requires urgent medical management. Approximately 80% of the patients benefit from initial therapy and mortality rates after treatment are 10-15% [6]. Current treatments include plasma exchange, adjuvant steroids, rituximab, and treatment of the associated complications [7]. Caplacizumab is a novel therapeutic agent that inhibits the interaction between v-WF and platelet glycoprotein-Ib [8]. COVID-19 emerged in 2019 and had an enormous global impact. COVID-19 infection has several hematological manifestations, and the entire pathogenesis remains to be elucidated. Acquired TTP can be caused by common triggers such as viral infections, human immunodeficiency virus, pregnancy, and certain immunosuppressants [9]. In this review, we describe a case of COVID-induced TTP and give a brief overview of the condition. This review raises awareness so that there is early recognition of any hematological manifestation associated with COVID-19, reducing the morbidity and mortality associated with the disease.

## Case presentation

**Figure 1:**
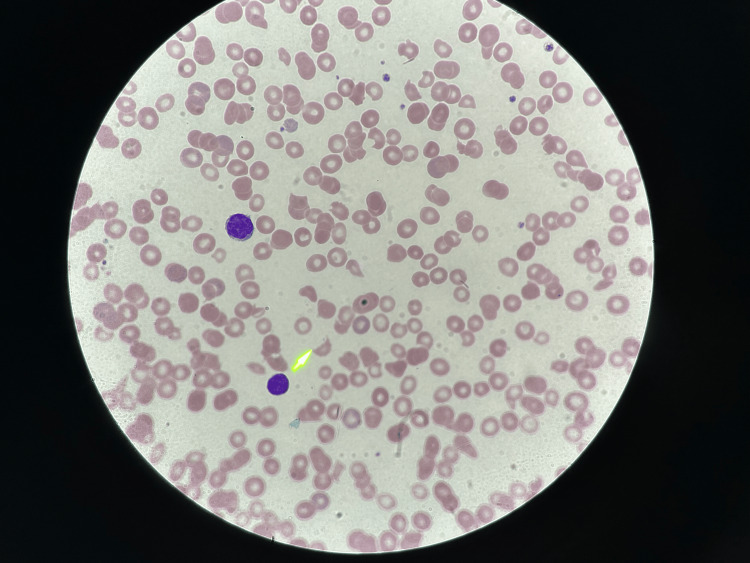
Peripheral blood smear Peripheral blood smear showed four to five schistocytes per high power field (HPF). The yellow arrow marks the schistocyte.

**Figure 2:**
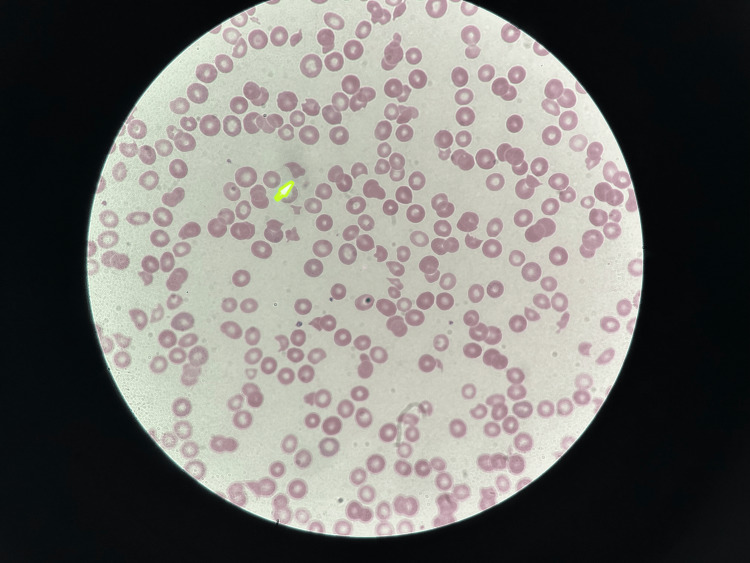
Peripheral blood smear Peripheral blood smear showed four to five schistocytes per high power field (HPF). The yellow arrow marks the schistocyte.

A 51-year-old female with a past medical history of hypertension presented to the emergency department (ED) with a complaint of dizziness and lightheadedness that started earlier in the day. She had recently visited urgent care for headaches and weakness and was diagnosed with COVID-19 a week ago. However, her symptoms were not improving so she decided to visit the ED. On her way to the hospital, she had right arm numbness. In the ED, the patient said she felt better and denied any numbness in the right arm. She did state she had a decreased appetite. She was afebrile, had a blood pressure of 183/91 mmHg, and a heart rate of 120 beats per minute. CT head showed no acute pathology and electrocardiogram (EKG) showed normal sinus rhythm. Her hemoglobin being 5.7 g/dL, she was given two units of packed red blood cells (PRBC) in the ED and two 2-liter fluid bolus. She also received meclizine for dizziness, and her potassium was repleted. A digital rectal exam (DRE) was done and revealed brown stools. Peripheral blood smear showed polychromasia, decreased platelet count with the absence of clumps, giant platelets, and four to five schistocytes per high power field (HPF) as shown in Figure 1 and Figure 2.

Given the anemia, thrombocytopenia, schistocytes on peripheral smear, acute kidney injury, low haptoglobin, lactate dehydrogenase (LDH) over 900 U/L, mildly elevated total bilirubin, and neurological symptoms on presentation; the clinical suspicion for TTP in the setting of COVID-19 infection was high. The plasmic score was 6 (high risk). The patient started on steroids (1 mg/kg prednisone) daily the next morning. ADAMTS13 activity and ADAMTS13 antibody were sent.

The patient was started on therapeutic plasma exchange (TPE) the next morning. Peripheral blood smear was reviewed daily, and it showed a slight improvement in schistocytes. Hemoglobin improved to 8.7 after the PRBC infusions. D-dimer of 2698 ng/mL, ADAMTS13 activity of <2%, and ADAMTS13 antibody of 21 units/mL were noted. LDH trended down from 930 units/L to 500 units/L. Platelet count also improved to 70 K/L after the first session of TPE. After four sessions of TPE (Days 2, 3, 4, and 7 of admission), ADAMTS-13 activity improved to 41.7%. During the hospital stay, she got a total of four plasmapheresis and the trend of lab values is shown in Table 1.

**Table 1:** Trend of patient's complete blood counts

	Normal value	Day of admission	After fresh frozen plasma, two packed red blood cells & first plasmapheresis	After the second plasmapheresis	After the second plasmapheresis	After the third plasmapheresis	After the fourth plasmapheresis
White blood cell count	4.80-10.80 K/uL	12.70	15.11	15.34	19.91	18.69	15.88
Red blood cell count	4.20-540 M/uL	2.62	3.61	3.02	3.55	3.21	3.56
Hemoglobin	12.0-16.0 g/dL	5.7	8.7	7.2	8.6	7.8	8.7
Hematocrit	37.0-47.0%	19.5	28.3	24.4	29.0	25.9	29.3
Mean corpuscular volume	81.0-99.0 fL	74.4	78.4	80.8	81.7	80.7	82.3
Mean corpuscular hemoglobin	27.0-31.0 pg	21.8	24.1	23.8	24.2	24.3	24.4
Mean corpuscular hemoglobin concentration	32.0-37.0 g/dL	29.2	30.7	29.5	29.7	30.1	29.7
Red cell distribution width	11.5-14.5%	29.6	27.0	26.6	25.9	24.9	23.2
Platelet count	130-400 K/uL	69	70	179	298	419	624

**Figure 3:**
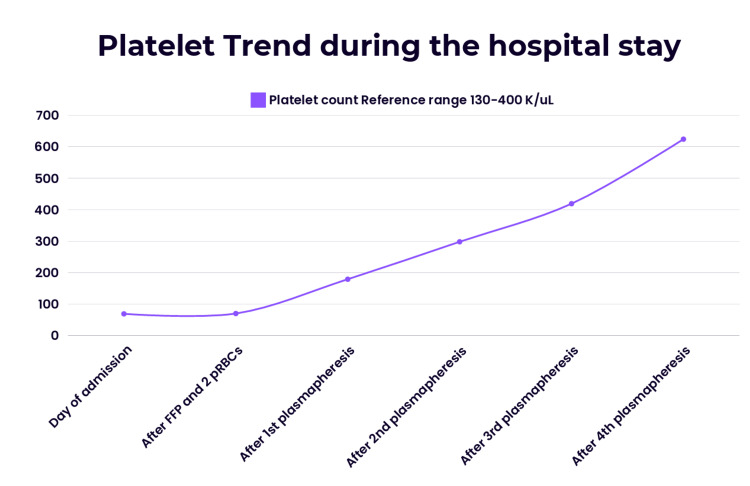
Trend of patient's platelets during the hospital stay

The platelet counts normalized, and LDH was slightly elevated but improved. Her platelet trend is shown in Figure 3.

She was started on rituximab on the third day of admission. Eventually, steroids were decreased to 60 mg per day, and tapering continued. The platelet count remained stable and was 649K/uL before discharge. LDH levels also improved, the plasma exchange catheter was removed, and she was advised to follow up with hematology as an outpatient.

## Discussion

Von Willebrand Factor (VWF) is a protein expressed on the endothelium and participates in platelet adhesion. When subjected to the shearing force from high blood flow conditions, VWF changes conformation to facilitate clotting. TTP results from a functional deficiency of ADAMTS13, a metalloprotease responsible for cleaving von Willebrand Factor (VWF) multimers. This causes an accumulation of large VWF multimers, and subsequently unchecked platelet adhesion and microangiopathic clot formation [10]. TTP is a life-threatening condition as per literature, various viruses play a role in the pathogenesis of this condition. DNA viruses, such as cytomegalovirus (CMV), cause TTP by causing injury to the endothelium and cytokine storm, whereas certain RNA viruses like HIV and influenza produce ADAMTS13 inhibitors and cause injury to the endothelium [11]. COVID-19 infection can trigger TTP, and it is hypothesized that the increased inflammation during the infection can lead to activation of the microvascular endothelium leading to TTP [12]. Another article describes that hypercoagulability in COVID-19 patients leads to a mild to moderate decrease in ADAMTS13 activity [13,14]. Today, the classic pentad of signs described in the literature, including fever, impairment of mental status, anemia, thrombocytopenia, and renal failure, appear in less than 10% of cases [3]. Our patient also had numbness of the arm, dizziness, acute kidney injury (AKI), and thrombocytopenia on presentation. A correct diagnosis for TTP is necessary, as it is fatal in 90% of patients if left untreated [15].

TTP is primarily treated with daily therapeutic plasma exchange (TPE), which is initiated emergently, without confirmatory testing of ADAMTS13 activity. Treatment is continued until a clinical response is achieved, defined as platelet stabilization >/= 150 K/uL, and lactate dehydrogenase resolves to <1.5x the upper limit of normal. Patients are said to have achieved clinical remission if they sustain this response for >30 days after completing TPE [16]. Immunosuppressive therapy is used concurrently with TPE for acute TTP management. Usually, prednisone 1 mg/kg is recommended [17]. Patients with more severe symptoms are treated with intravenous methylprednisolone. Our patient was treated with prednisone 1 mg/kg.

The American Society of Hematology recommends the use of rituximab (monoclonal chimeric antibody against CD20) in TTP. However, it should be delayed till the acute infection has resolved or there is production of COVID-19 antibodies [18]. We also started our patient on rituximab a few days after her admission and post-plasmapheresis. A recent meta-analysis showed rituximab has been shown to improve relapses and mortality during acute iTTP episodes [19]. As TTP carries high mortality rates, prompt treatment is necessary. Since there was high suspicion for TTP, the patient was empirically started on high-dose steroids and plasmapheresis. Caplacizumab is a novel agent that inhibits the interaction between vWF and platelet glycoprotein Ib [8] and is also used in COVID-induced TTP. Caplacizumab is a bivalent anti-VWF immunoglobulin approved in 2019 for the treatment of acquired TTP, for use in conjunction with TPE and other immunosuppressive therapies. In the Phase II Clinical Trial TITAN, caplacizumab co-administered with TPE was found to have a 39% reduction in time to achieving response, when compared to the placebo group of TPE alone [20]. In the trial, caplacizumab was initiated as a 10 mg intravenous (IV) dose on Day 1 of TPE treatment, which was then given daily before TPE as a 10 mg subcutaneous dose and was continued for 30 days following cessation of TPE. Only 8% of patients in the treatment arm experienced exacerbations, compared to 28% of patients who received the placebo [21]. Notably, during the overall trial period, 12% of patients in the caplacizumab-treated group had a relapse as compared to 38 % in the placebo group. The adverse effect reported with caplacizumab is mucocutaneous bleeding [21]. Refractory TTP is when platelets fail to respond to plasma exchange and immunosuppressants after four to seven days or worsening of the symptoms despite being on treatment for seven days. It is challenging to manage refractory TTP. Vincristine has been used as an adjuvant to TPE for the management of refractory TTP, however data are fairly limited [22]. Splenectomy is considered in refractory cases. Our patient improved after plasmapheresis and was advised to follow up with hematology as an outpatient with closer monitoring of her CBC and ADAMTS13 levels.

## Conclusions

There is growing evidence that COVID-19 can trigger TTP, and prompt diagnosis is crucial. Elucidation of the mechanism and optimal treatment are still under research. The management is challenging, and a multimodal approach is required for better outcomes. Additional prospective clinical trials are required for a better understanding of the treatment and the use of plasmapheresis during COVID-induced TTP. Due to the unpredictability of COVID-19 and its complications, robust research is needed to understand the mechanism and determine which patients are more at risk for adverse outcomes.
